# YIM-O13. Development of novel protein biomarkers for the prediction of response to treatment in juvenile idiopathic arthritis

**DOI:** 10.1186/1546-0096-12-S1-Y1

**Published:** 2014-09-17

**Authors:** Jason Palman, Simona Ursu, Wendy E Heywood, Kevin Mills, Lucy R Wedderburn

**Affiliations:** 1 Infection, Immunity, Inflammation and Physiological Medicine Programme, Infection, Inflammation and Rheumatology Section, Institute of Child Health, London, UK; 2 Centre for Translational Omics, Institute of Child Health, London, UK

## Introduction

Standard management of Juvenile Idiopathic Arthritis (JIA) is heavily dependent on the use of methotrexate (MTX), which induces clinical benefit in about 65% of patients. We have previously demonstrated that children with high serum levels of MRP8/14 protein prior to starting MTX have a high chance of good response to MTX. However, among children whose MRP8/14 serum levels are moderate or low, prior to MTX, some respond well whilst others respond poorly to MTX. We have taken a proteomics approach to identify novel biomarkers present in serum prior to treatment that correlate with response or non response to MTX.

## Objectives

The aim of this study was to identify novel protein biomarkers in serum from JIA patients to help and inform the clinicians at time of diagnosis whether the patient is likely to respond well to MTX.

## Methods

Blood serum samples and clinical data were obtained from 83 JIA patients from the Childhood Arthritis Response to Medication Study (CHARMS) before starting MTX treatment. The study has full ethical approval and consent. To assess clinical response to medication, core set variables and Definition of Improvement (DOI) for JIA was used, comparing data at 0 and 6 (range 4-8) months. MRP8/14 was measured in these patients and used to classify cases into three groups: non-responders, responders with low MRP 8/14 levels pre MTX, and responders with high MRP 8/14 levels pre MTX. To screen for proteins which distinguish future MTX non-responders from responders, 5 serum samples from age-matched patients from each group were pooled; pooled samples were enriched for low abundant proteins, and fractionated on a 1D PAGE. Proteins were identified and quantified by Mse- label free quantitation and analysed using Nonlinear Dynamics Progenesis LC-MS software for differential expression.

## Results

A total of 648 unique proteins were identified in serum pools (just prior to commencing MTX treatment). Patients were defined as responders, R (those reaching ACR50 or above) or non-responders, NR (reaching only ACR30 or below). Forty proteins were found to be differentially represented in the serum pools, using a level of >2 fold difference between MTX responders and non-responders. This group included 26 proteins with high abundance in the responder group and 14 proteins with high abundance levels in the non-responder group. Using a more stringent cut off of fold difference > 4 identified 12 proteins that showed a significant difference between R and NR groups. In contrast to our previous data with MRP8/14, these 12 novel biomarker proteins were highly differentially expressed in serum of both the groups of responders (i.e. MRP high and MRP low responder groups), compared to NR cases, prior to MTX use. This indicates that these protein biomarkers may function to distinguish R from NR patients more efficiently than the MRP8/14 biomarker.

## Conclusion

This study has identified a number of potential novel protein biomarkers which may be able to discriminate between patients who will respond or those who will fail to respond to MTX, when measured prior to starting the treatment. Forty protein biomarkers were able to significantly differentiate between response and non response. Using a higher cut off of >4 fold difference produces 12 proteins that we envisage to be more reliable than MRP 8/14 for predicting response. Further testing and analysis of these proteins is required to validate those which can most reliably recognise patients who will respond to MTX therapy, or those who will fail to respond.

## Disclosure of interest

None declared

